# Integrating Patient‐reported Experience (PRE) in a multistage approach to study access to health services for women with chronic illness and migration experience

**DOI:** 10.1111/hex.13649

**Published:** 2022-11-23

**Authors:** Thomas Abel, Lidya Tadesse, Annika Frahsa, Sibel Sakarya

**Affiliations:** ^1^ Institute of Social and Preventive Medicine (ISPM) University of Bern Bern Switzerland; ^2^ Johns Hopkins Bloomberg School of Public Health Baltimore Maryland USA; ^3^ Department of Public Health, School of Medicine Koç University Istanbul Turkey

**Keywords:** agency, health services, immigrant women, inequity, methodology, patient experience

## Abstract

**Background:**

Patient‐reported Experience (PRE) is an emerging concept integrating patient perspectives and amplifying voices often marginalized in discussions surrounding health systems. However, it remains a challenge to use and integrate PREs when studying patient agency and access to quality services, particularly with data from multiple sources. In this article, using study materials from the Swiss MIWOCA project, we present and reflect upon a multistage PRE approach to study healthcare access.

**Methods:**

The MIWOCA project, a study on healthcare access and quality among immigrant women with chronic illnesses living in Switzerland, provided data from multiple sources for the integration of PRE data. These sources included interviews with women (*n* = 48), two focus group discussions with women (*n* = 15), interviews with service providers (*n* = 12) and observations from stakeholder dialogues (*n* = 3). In addition, we utilized field notes, focus group illustration maps, patient vignettes and policy briefs to develop a multistage data linking model. PRE data served as starting themes and reference topics in each of the interlinked stages of knowledge production.

**Results:**

Deploying PREs, we coherently linked the data from preceding stages and used them to inform subsequent stages. This, in turn, enabled us to identify, reflect and rectify factors limiting immigrant women's agency and access to quality services. Ultimately, the approach engaged patients as knowledge co‐producers for system‐level changes. This knowledge was transformed into a set of practice recommendations and a policy brief addressing ways to improve health systems to better serve immigrant women in Switzerland.

**Conclusions:**

Building on PREs to systematically combine multiple data sources and engage patients continuously can improve our understanding of barriers in health systems. Beyond individual patient‐doctor encounters, a multistage PRE approach can identify structural problems and provide clues for resolving them at the systems level. The PREs approach presented may serve as an example and encourage more public health experts to consider PREs in future research and practice.

**Patient and Public Contribution:**

Women with chronic illness and immigration experience contributed to interview‐guideline development, provided PREs in interviews, identified priority areas for health‐service change and actively participated in the development of practice recommendations.

## 1 INTRODUCTION

Patient‐reported Experiences (PREs) are an emerging concept that addresses how patients view and interpret their interactions with healthcare systems. PREs reflect how patients perceive their access to and quality of care.^1^, ^2^ PREs are particularly valuable when considering interactions of various dimensions of care that can include (but are not limited to) patient satisfaction, patient perception, patient preferences and patient engagement.^3^ Although broad in their scope, most current PRE approaches focus on their potential for improving the individual healthcare experience in clinical settings, during or within a patient's encounters with services.^4^, ^5^

PREs are still in their early stages of conceptual development and remain a topic of wide discourse amongst health researchers and professionals. Many issues are currently on the research agenda, including the appropriateness and effectiveness of current methods in creating and implementing patient‐reported experience measures (PREMs).^6^, ^7^, ^8^ Currently, most studies exploring PREs/PREMs use quantitative approaches. This is particularly true for leading health systems that have implemented PREs/PREMs into service evaluations; primarily in the form of practice‐specific questionnaires.^9^, ^10^ However, using questionnaires to capture PREs has its limitations, especially in that survey methods and data can be difficult to administer and interpret for healthcare staff, which acts as a significant barrier to their wider use.^11^ Moreover, standardized quantitative methods are limited in addressing complex issues related to patients' utilization patterns. This includes questions on how system features affect patients' perception, knowledge and ultimately their behaviours.^12^, ^13^

A particular challenge for PRE approaches is addressing social inequalities stemming from structural disadvantages. Many health system evaluations demonstrate that patients' access to high‐quality healthcare services varies significantly by their social backgrounds (i.e., gender, race, migration status, socioeconomic status, etc.). A 2019 report on social inequalities in health systems found that across all OECD countries, Indigenous peoples and ethnic minorities were more likely to face socioeconomic disadvantages, language problems, cultural barriers and discrimination—all of which increase the likelihood of experiencing health disparities.^14^ Qualitative PRE approaches may better capture these issues, particularly those of intersectionality, which is important to understand in patient groups experiencing multiple disadvantages.^15^, ^16^ Moreover, as these population groups also often face a higher burden of disease, their involvement in working towards improving services is even more crucial.^17^

Given this background, the present paper demonstrates how a PRE approach can be used to link various data sources addressing healthcare system shortcomings. In doing so, we draw on data and insights from an empirical research study (Migrant Women's Health Care Needs for Chronic Illness Services in Switzerland [MIWOCA]) on immigrant women's healthcare needs and access to chronic illness care.^18^ In the current paper, we illustrate how a multistage qualitative methodology can integrate patients' experiences in consecutive steps of the knowledge‐production process and engage patients as co‐producers of knowledge for improving care services.

## 2 METHODS

This is a sub‐study of MIWOCA, short for Migrant Women's Health Care Needs for Chronic Illness Services in Switzerland (SNF NRP74 2017‐2020), a larger research project in which we researched access to and quality of healthcare service among women in Switzerland with chronic illness and migration experience.^18^, ^19^ MIWOCA included migrant and native‐born women with chronic illnesses across diverse cultural and social backgrounds as well as care providers and other relevant stakeholders. Women participated in interviews and focus group discussions (FGDs). Subsequently, they were invited to participate in a series of stakeholder dialogues convened to develop policy recommendations. Detailed information about the study population, sampling strategies and data collection has been described previously.^18^ In the current paper, we present a methodological approach to linking data sources based on the empirical fieldwork conducted during the MIWOCA project. We used data from multiple sources: observations and field notes from regular project meetings and three stakeholder dialogues, an analysis of project documents (such as minutes, reports, notes, patient vignettes, MIWOCA evidence and policy briefs), as well as a re‐analysis of qualitative semi‐structured interviews with women with chronic illnesses and health/social service providers (*n* = 48; *n* = 12) and two FGDs (*n* = 15). A survey among study participants provided auxiliary data on participants' perception and assessment of the multistage approach.

To develop our data linking model, we analysed field notes, documents and interview and focus group data, the latter ones had been analysed using the framework method,^20^ supported by Atlas.ti software. We applied the following steps: transcription, familiarization with the data, coding and categorizing, identifying themes, developing a working analytical framework, applying an analytical framework, charting the data into the framework matrix and interpreting the data. We used both inductive and deductive approaches to help create an analytical framework. Throughout the data analysis process, we held regular peer debriefings among co‐authors to discuss and refine findings and to interpret the meanings of data.

## 3 RESULTS

In the following, we present (1) the multistage approach from MIWOCA that integrates PRE data, and thus patient involvement, at each step. We also present (2) selected examples of specific PREs on access to and quality of healthcare services. We will use those concrete examples to demonstrate how PREs were continuously reflected and considered throughout the multistage methodology to ultimately generate recommendations for improving access to and quality of healthcare services for this patient population. In addition to that, we present (3) participants' perceptions of the multistage PRE approach developed here.

### 3.1 The multistage approach to integrating PREs

**Figure 1:**
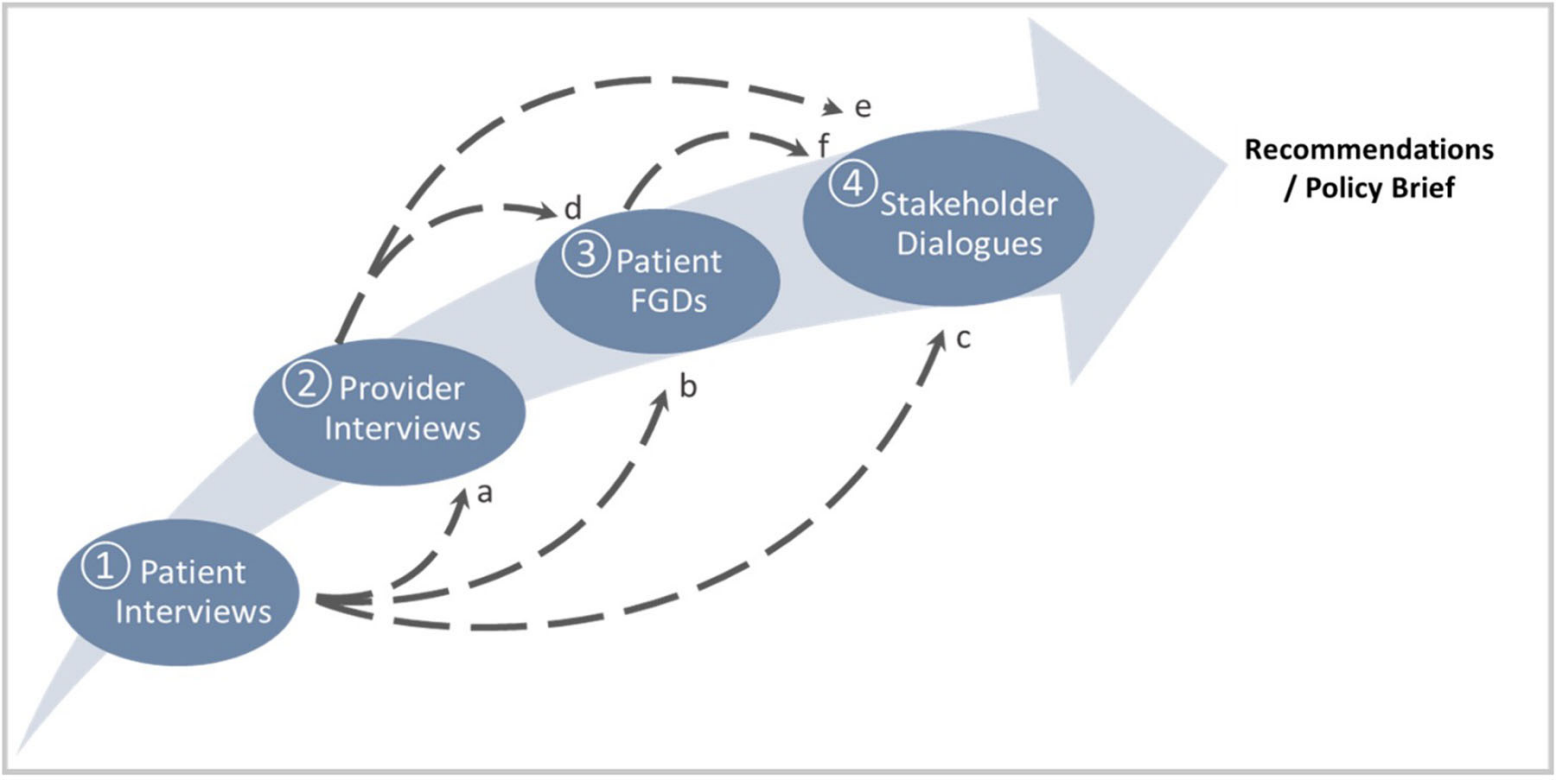
Data flow in MIWOCA. Six data streams linking four different sources. In streams a–c, PREs directly informed the next step in data production and analysis. In streams d–f, PREs merged with other data to inform the next step in data production. MIWOCA, Migrant Women's Health Care Needs for Chronic Illness Services in Switzerland; PRE, Patient‐reported Experience.

Figure 1 depicts how PREs were synthesized, analysed and applied between these main stages: (1) interviews with patients, (2) interviews with providers, (3) FGDs with patients and (4) stakeholder dialogues. In each stage based on a different data source, researchers applied findings from previous stages to inform subsequent steps.

In Stage 1, researchers conducted interviews with patients. Findings from these interviews informed the themes addressed in interviews with providers in Stage 2 (stream a). These findings were also used in later parts of the study (streams b and c). Interviews in Stage 2 allowed researchers to contrast perspectives between patients and providers and identify structural factors that impact access to and continuum of care. Findings from interviews with patients and providers informed the themes addressed in FGDs with patients in Stage 3 (streams b and d). In FGDs, patients reflected upon these findings and presented their views on priority topics to be discussed in stakeholder dialogues (Stream f). In Stage 4, researchers conducted stakeholder dialogues that incorporated diverse perspectives from relevant parties, including those from previous steps (streams c, e and f). Ultimately, the key findings generated in this data flow were used to formulate and finalize a set of practice recommendations for health professionals and policymakers. The recommendations were later included in a policy brief and distributed among key actors in Swiss healthcare policy and organizations (see Supporting Information: Additional Files 1 and 2).

We used findings from data analysis in different formats: PREs from Stage 1 were used as substantive findings that were analysed and reported (e.g., identified barriers to accessing particular healthcare services [lack of system knowledge, stigma, etc.]). PREs from Stages 1 and 3 were also used to develop complementary formats, namely ‘vignettes’ and ‘focus group illustration maps’.^21^ These formats aimed to represent ‘thick descriptions’^22^ of patients' experiences and were presented and discussed as part of the stakeholder dialogues in Stage 4.

Each stage combined additional and different kinds of data. As a consequence, the increased richness and complexity of this data facilitated data triangulation and advanced analyses. Likewise, findings from each stage served to inform the content and focus of the next stage. For example, parts of the analysis of interviews with patients were used to help create the interview guide for interviews with providers. In principle, insights from PREs were carried forward to each mode of data production and analysis. In integrating data from all stages, we identified barriers and resources to accessing care (e.g., women's unfamiliarity with the complex healthcare system; informal social support networks). These were confirmed when merging the different sources, including other patients, care providers and third‐party stakeholders (e.g., insurance experts). Through applying this model, we were able to gain insights on (1) why and how accessibility barriers are linked to specific healthcare utilization behaviours and limitations in the patient agency, (2) how such patterns are distributed among migrant women with chronic illnesses and (3) how these factors and processes can be improved.

### 3.2 Specific PREs and their integration throughout the multistage approach

#### 3.2.1 PREs identified from interviews with patients

PREs often addressed women's knowledge of the health system's available health services and how to access those services, health insurance options and issues of how to mobilize social support. Those issues were identified as key elements for a patient's ability to make choices and use health services effectively thus, facilitating their agency. Here, the patient agency is defined as the ability of patients to influence and contribute to the decision‐making process behind their care.^23^

Through PREs, women interviewed provided rich context information yielding a better understanding of issues such as limited health knowledge among certain population subgroups or lack of financial resources needed to utilize the healthcare services. In regard to the former, women described how their perspectives relate to both sides of the provider–patient relationship, specifically in the context of receiving and understanding medical information. On the one hand, patients critically evaluate the quality of information providers offer. On the other hand, they also reflect on their positions as receivers of this information. Patients highlight issues such as the lack of information, lack of clarity of the information or lack of details. For example, one woman notes her disapproval:

It's the 8th day now, but how aware am I [about the medication]. How much information was given to me? They didn't provide information about anything; this is used for these diseases; this is used for these people … do you really want it 100% or not … they didn't say that.

Apart from direct interaction between medical service providers and patients and its impact on knowledge of medication, the women interviewed provided context on the reasons why they do not have complementary health insurance. One patient explains:

And it's not that I don't trust the public health care system that I want a complementary plan. For a long time, I didn't have any complementary insurance because I had trusted the hospital so much. I have always found the quality of care to be good.

Participants also expressed a need for better knowledge about navigating the health system and how this lack of knowledge ultimately limits their abilities to access health services and make informed decisions. For example, one participant noted:

There [should] be more openness. And that this support [should] be made available in several languages, not only German […] when I arrived in Switzerland, it was ‘and now, what doctor am I going to?’ The options are reduced. So sometimes I ended up picking a doctor just because they spoke English.

These examples highlight how PREs help to address ‘how’ and ‘why’ questions on potentially widely known aspects, such as alleged health illiteracy and lack of financial resources.

#### 3.2.2 PREs applied in the providers' interviews

To facilitate the utilization of different data sources and further explore those issues around information deficits, language problems and access to care, researchers included the following questions in the interview guide for interviews with providers (*Stage 2*):

(1) Do you think the current healthcare system works equally well for
  (i) women compared to men with chronic illnesses?
  (ii) For Swiss women compared to foreign‐born women?
  (iii) For women with lower versus higher educational attainment?
(2) Are there any consequences of those differences?

Providers echoed not only that immigrant women with chronic illnesses have less information on how the health system operates, but also that this negatively impacts the ways in which they access care. For example, one provider stated:

If you put literacy problems, language barrier issues, lack of knowledge of the health care system, fear of seeking care […] How many people are not seeking care! […] In relation to insurance too, it's sometimes complicated to understand […] It can be a little complicated sometimes […] Knowing what you're doing, what you need to do…

Likewise, another provider (based in the French‐speaking part of Switzerland) noted:

Yes, I think [it's complicated]. Yes, especially for a non‐French‐speaking woman. It's complicated. For me, the ones I see, they were helped, e.g., by a friend who spoke French. But how else do they call, find a phone number, make an appointment, know where to go? It's complicated […]

#### 3.2.3 PREs applied in the FGDs

Researchers synthesized and analysed initial findings from Stages 1 and 2 in a short report and presented this to immigrant women (streams b and d) participating in FGDs (*Stage 3*). During FGDs, these issues acted as three stimuli to the participants: (1) access to health care, (2) interactions with healthcare providers and (3) potential solutions to problems as proposed by interview participants. More specifically, researchers presented condensed findings from previous stages. We used a knowledge mapping approach^21^ to visualize discussion results and to have participants prioritize issues by assigning points to them.

Participating women reported three key demands for change at the system level:

(1) better access to transcultural communication with healthcare providers,
(2) more support and counselling on the day‐to‐day struggles of managing a chronic illness,
(3) and early access to user‐friendly information about health insurance, particularly policies and contracts.

#### 3.2.4 PREs applied in the stakeholder dialogues

Finally, stakeholder dialogues were conducted (*Stage 4*). Findings from the previous stages were synthesized into a MIWOCA brief and vignettes (cf. Supporting Information: Additional Files). Vignettes addressed (1) communicative competence in terms of language, culture and lifestyle, (2) being understood without prejudice, (3) transparent communication and adequate information about basic insurance policies, and (4) information about supplementary insurance policies, especially for those new to Switzerland. Vignettes would be read out aloud to make findings more amenable and palpable. They were orally presented during the stakeholder dialogues, as in the following:

She is also not satisfied with her current insurance, she complains. The tall woman, wrapped in countless layers of sweaters and cardigans, pulls out a considerable stack of documents. She immediately adds that she printed out all these files in the office ‘on a private budget, of course!’ They are documents from a wide variety of health insurance companies, all of them annotated. ‘How can anyone wade through this insurance jungle? Where is the best offer for me, both in terms of the costs to pay and the services I need with my illness?’ she laughs somewhat helplessly. (Excerpt from patient vignette, presented in the stakeholder dialogues, cf. Supporting Information: additional file 3)

Discussing the findings in the stakeholder dialogues, participants recognized that, while immigrant women had good self‐perception and self‐efficacy concerning individual health, practitioners and policymakers should prioritize improving immigrants' health system knowledge and the accessibility of health system information. Moreover, they acknowledged this would improve patients' overall agency.

Last, participants transformed the findings from the stakeholder dialogues into a list of final recommendations to be distributed to relevant parties. To give just one example: In addressing immigrant women's lack of access to relevant health system information specifically, these recommendations included the following:

Low‐threshold information services should be promoted in communities and neighbourhoods. Communities should offer orientation aids for patients with chronic diseases at the neighbourhood level, especially for: the search of social services, health care services, and self‐help groups; navigation in complex systems (e.g., adequate insurance models and services); questions concerning patient rights. (cf. more detail additional files)

The recommendations were thus developed including the initial PREs, advanced in various stages. They were afterwards transformed into a policy brief on the access to and quality of health care for immigrant women with chronic illnesses living in Switzerland (see Supporting Information: Additional File 2). This was systematically distributed via media relations of the University of Bern and the MIWOCA project webpage.

### 3.3 Study participants' perspectives on the multistage approach

Study participants varied in their perception of the multistage approach to integrating PREs. Participating women tended to express a great level of enthusiasm for engagement as well as gratitude ‘for being really heard’ as expressed by one woman. At the same time, some women discontinued participation or voiced disappointment about the scope of the project over time. They had hoped to receive individually tailored recommendations for their own health and context, concrete advice concerning their own situation rather than to contribute to a system‐level discussion and recommendations.

Participating service providers stated that it is crucial but nevertheless not very common in their contexts to involve people concerned from the beginning and in all phases of the research. However, some participants also expressed doubts about the usability and interpretation of PREs beyond an individual patient's experience in a broader context.

I think it is important to focus on the patients' experiences for once. However, this approach leaves it somewhat open whether these are individual cases or whether these experiences can be generalized. Interpretation is also difficult because we only know the view of those affected. But basically, an exciting approach! (Response in the follow‐up survey of the stakeholder dialogue)

## 4 DISCUSSION

This study presented an example of how PRE data can be linked and utilized in health services research. PREs from women with chronic illnesses formed the core of data collection, linking and analysis, and in the development of practice recommendations. PRE data from multiple sources were integrated into multiple stages to produce a more comprehensive understanding of the conditions, factors, and processes impacting patients' agency in access to care services. In a different function, PREs from interviews and FGDs served to focus and complement perspectives gathered through interviews with providers, jointly offering a more comprehensive view of systemic and institutional factors affecting the quality of services. Moreover, PREs functioned as trigger points and guided the agenda during stakeholder dialogues. In presenting PREs to health experts who participated in these dialogues, PREs finally facilitated meaningful discussions on how relevant parties can improve the health system to provide better care for immigrant women with chronic illnesses. While PREs varied in their function and formats over the course of the study, they remained the driving force behind this data flow and the knowledge base used to formulate final recommendations.

The multistage qualitative approach combining multiple data sources allowed to concretely involve patients in exploring existing problems and give them a voice in creating solutions. From a wider perspective, the use of the PREs methodology presented here aligns with the concept of co‐production, which Greenhalgh et al.^24^ define as, ‘the collaborative generation of knowledge by academics working alongside stakeholders from other sectors’. In the current approach, co‐production of knowledge allowed researchers to identify systems‐level issues while maintaining a focus on the actual lived experiences of patients.^24^ The use of PREs exemplified how they can be applied to capture more of the complexity that emerges from the manifold social and economic challenges typically seen in immigrant populations, especially those facing chronic health problems. Within the context of immigrant health specifically, linking data from different sources while keeping a focus on PREs can help researchers better understand the challenges and barriers that are otherwise difficult to identify (e.g., language problems, lack of familiarity with health and social care services, effects of discrimination, etc.).

In the case of Switzerland, examples of these obstacles include challenging administrative conditions, complicated health and social insurance schemes and difficulties in communicating with care providers in the absence of translation services. It is likely that other countries face similar challenges, and that PRE research can help to describe and understand such structural conditions and how they interact.

The multistage approach of integrating PREs was developed with a focus on chronic illness care and included women living in Switzerland most of them with a migration background. This poses limitations for transferring the approach to other healthcare settings. However, the basic approach of using PRE in participatory research on structural conditions and patients' agency can still be feasible in different contexts‐adjusted, though, to each country's unique conditions. As the current paper's primary focus was to introduce a new methodological approach that builds on the systematic linking of different data sources, it could not address the pending issue of how to best assess PREs in different contexts. The current use of PREs is often disease‐ or area‐specific.^9^, ^10^ The findings presented here indicate that a more needs‐ and context‐specific use of PREs may contribute to a more differentiated understanding of the interplay of the structural conditions and patient agency—that is, PREs for specific populations or with specific needs in mind. Thus, the current methodological approach might aid researchers and practitioners in developing new measures, which are particularly useful for evaluating health services. Last, PRE approaches can and should consider other current developments in the field, namely those focusing on patients as social actors in and for their health. The approach presented here aligns with current research on people‐centred services, which the WHO defines as care that emphasizes the ‘[…] perspectives of individuals, families and communities’, and views people as ‘[…] participants as well as beneficiaries of trusted health systems’.^25^ Moreover, the use of PREs in advancing people‐centred care will allow institutions to identify problems with the delivery of care, implement new changes and interventions based on patient feedback and might promote the transparency and accountability of healthcare providers.^26^

As researchers in the current study integrated perspectives from various stakeholders, this did not come without limitations. Although patient engagement was present in every stage of the knowledge‐building process, patients' personal participation declined while moving from identification of care deficits to analysing the data and drafting recommendations in stakeholder dialogues. In addition, women who were rather fluent in German tended to be overrepresented when it comes to continuous participation, despite translation services offered at meetings and dialogues. At the same time, the women who continuously participated in the MIWOCA study had been in close interaction with other patients through the group discussions and perceived themselves as representatives, particularly for the ones who might not have felt ready to speak up. Additionally, while stakeholder dialogues included representatives from a wide variety of sectors, future studies might consider including patients' families and caregivers as they can provide unique perspectives on relevant topics.

## 5 CONCLUSION

Integrating patients' experiences proved a useful approach in the current system‐oriented research on access to health services. Applying a multiple‐stages process that featured PRE data allowed us to successfully link different sources and formats of data. This integrative PRE approach nurtured a process of co‐production allowing to engage chronic disease patients in each step of the research process, from the identification of problems to the development of recommendations to mitigate them. While the focus was on women with a migration background in Switzerland, the methodology presented may facilitate future PRE studies reaching beyond the themes and contexts addressed here. The method presented may thus serve as an example and encourage more public health experts to consider a PRE approach for patients' involvement in health systems research.

## AUTHOR CONTRIBUTIONS

Thomas Abel defined the main research question, developed the structure of the paper, wrote major parts of the text and lead the author team. Lidya Tadesse conducted the literature search and its integration into the text, contributed to creating a focus for the argument and wrote major parts of the text. Annika Frahsa contributed to the development of the argument, analysed and interpreted data sources and wrote major parts of the methodology description. Sibel Sakarya helped in developing the focus and the structure of the paper. All authors contributed to the revision of the manuscript. All authors read and approved the final manuscript.

## CONFLICT OF INTEREST

The authors declare no conflict of interest.

## ETHICS STATEMENT

We submitted this study to the Ethics Commission of the canton of Bern who determined no formal ethics approval was needed. All participants provided written informed consent, except for two women, who instead provided oral consent.

## Supporting information

Additional File 1.

Additional File 2.

Additional File 3.

## Data Availability

The data sets generated and analysed during the study cannot be shared as participant confidentiality could be compromised if full interview transcripts were released.
