# Supplementary material for: Integrating Patient‐reported Experience (PRE) in a multistage approach to study access to health services for women with chronic illness and migration experience
Source: Health Expect. 2022 Nov 23;26(1):237–44. doi: 10.1111/hex.13649 (PMC9854289; doi:10.1111/hex.13649)
Supplement: Supplementary file 1 — Additional File 1. [file HEX-26--s003.docx]

# Add. File 3: German and English version of a MIWOCA vignette

# Story2 39 year old German woman

When we ask her about her everyday life and whether she would like to describe how we can imagine it, Kathrin takes a deep breath, furrows her brow and thinks about it for a moment. "Well, it really only has two or three fixed points that always remain the same. Everything else then depends on the acute pain or the relapses." She slowly stretches up one finger after the other: "The fixed points are taking the medication, driving to work, although that is also influenced by my condition, and, more tediously, making phone calls to the doctors every day." She explains that it is precisely this juggling of work and doctor's appointments that costs her a lot, both energy and money. She asks us with tired eyes if someone else can keep an eye on the whole thing, and it seems as if all the hours of phone calls and clarifications with doctors and therapists speak for themselves. "With my part-time job at the library, I can't make any big leaps. If a massage appointment comes up, or the deductible for several treatments in a month, I have to take something from the rent account on short notice to pay the bills on time." The 39-year-old is lucky; her landlords are lenient and sometimes turn a blind eye. "It has also happened that they had to squeeze both eyes tightly shut, back when I had to have the knee operated on. I really had to scrape together the deductible from all kinds of sources."

She's also not happy with her current insurance, she complains. The tall woman, wrapped in countless layers of sweaters and cardigans, pulls out a considerable stack of documents. She immediately adds that she printed out all these files in the office "on a private budget, of course! They are documents from a wide variety of health insurance companies, all of them annotated. "How can anyone wade through this insurance Latin jungle? Where is the best deal for me, both in terms of the costs I have to pay and the benefits I need with my illness?" she laughs, somewhat helplessly. "And I can forget about supplemental insurance anyway," she says, circling her entire body with her hands in the air to signal where her health issues are, "no one wants to insure that." We end the conversation after about an hour; Katharina is motivated to continue working on our project in the future. "Maybe it will be possible to develop a strategy for some of my concerns in joint reflections?" she smiles at us and says goodbye to us into the meanwhile dark November night.

(translated with deepl.com)

# Story2 39jährige deutsche Frau

Als wir nach ihrem Alltag fragen und ob sie uns schildern möchte, wie wir uns diesen vorstellen können, holt Kathrin tief Luft, legt die Stirn in Falten und überlegt kurz. «Also der hat eigentlich nur zwei, drei Fixpunkte, die immer gleichbleiben. Alles andere hängt dann von den akuten Schmerzen respektive den Schüben ab.» Sie streckt langsam einen Finger nach dem anderen hoch: «Fix sind die Medikamente einnehmen, zur Arbeit fahren, wobei auch das von meinem Befinden beeinflusst wird und mühsamer weise auch alltäglich ist das Telefonieren mit den Ärzten.» Sie erklärt, es sei eben dieses Jonglieren von Arbeit und Arztterminen, dass sie viel koste, sowohl Energie als auch Geld. Ob nicht jemand ausser ihr selbst das Gesamte im Blick und im Griff haben kann, fragt sie uns mit müden Augen und es scheint als sprächen daraus all die Stunden von Telefonaten und Abklärungen mit Ärztinnen und Therapeuten. «Mit meinem Teilpensum in der Bibliothek sind grössere Sprünge nicht möglich. Kommt da einmal ein Massagetermin hinzu oder der Selbstbehalt mehrerer Behandlungen in einem Monat, muss ich kurzfristig vom Mietkonto was nehmen, um die Rechnungen fristgerecht bezahlen zu können.» Die 39-jährige hat Glück, ihre Vermieter sind nachsichtig und drücken manchmal ein Auge zu. «Es kam auch schon vor, dass sie beide Augen fest zudrücken mussten, damals als ich das Knie operieren lassen musste. Den Selbstbehalt musste ich wirklich aus allen möglichen Quellen zusammenkratzen.»

Sie sei zudem nicht zufrieden mit ihrer aktuellen Versicherung, beklagt sie sich. Die grosse, in schier unzählige Schichten von Pullovern und Strickjacken gehüllte Frau holt eine beachtliche Beige von Unterlagen hervor. All diese Dossiers habe sie sich im Büro ausgedruckt «natürlich auf privates Budget!», schiebt sie sofort nach. Es sind Unterlagen verschiedenster Krankenkassen, alle sind mit Notizen versehen. «Wie kann sich jemand durch diesen Versicherungs-Latein-Dschungel kämpfen? Wo gibt es für mich das beste Angebot, sowohl in Bezug auf die zu tragenden Kosten als auch auf die Leistungen, die ich mit meiner Krankheit brauche?» lacht sie etwas hilflos auf. «Und die Zusatzversicherung kann ich sowieso vergessen», meint sie und kreist mit den Händen in der Luft um ihren gesamten Körper, um zu signalisieren, wo sich ihre gesundheitlichen Baustellen befinden, «das möchte niemand versichern.» Wir beenden das Gespräch nach rund einer Stunde, Katharina ist motiviert auch künftig an unserem Projekt mitzuarbeiten. «Vielleicht wird es in gemeinsamen Reflexionen ja möglich, für einige meiner Anliegen eine Strategie zu entwickeln?» lächelt sie uns zu und verabschiedet uns in die unterdessen dunkle Novembernacht.
