# Supplementary material for: Integrating Patient‐reported Experience (PRE) in a multistage approach to study access to health services for women with chronic illness and migration experience
Source: Health Expect. 2022 Nov 23;26(1):237–44. doi: 10.1111/hex.13649 (PMC9854289; doi:10.1111/hex.13649)
Supplement: Supplementary file 2 — Additional File 2. [file HEX-26--s002.pdf]

UNIVERSITÄT BERN

MIWOCA
Migrant Women's Health Care Needs for Chronic Illness Services in Switzerland

74 NFP Gesundheitsversorgung Nationales Forschungsprogramm

10 EMPFEHLUNGEN ZUR VERBESSERUNG DER GESUNDHEITLICHEN VERSORGUNG VON FRAUEN MIT CHRONISCHEN ERKRANKUNGEN UNTER BESONDERER BERÜCKSICHTIGUNG VON PATIENTINNEN MIT MIGRATIONSERFAHRUNG

Die folgenden Empfehlungen basieren auf qualitativen Interviews und Fokusgruppen mit Patientinnen (die meisten davon mit Migrationserfahrungen), auf qualitativen Interviews mit Anbietern von Gesundheitsleistungen und auf strukturierten Diskussionen mit relevanten Interessensgruppen (Stakeholder Dialogen).

Die Empfehlungen richten sich an **Personen, die in der gesundheitlichen Versorgung, Pflege oder sozialen Unterstützung von Menschen mit chronischen Erkrankungen tätig sind**. Dazu gehören die entsprechenden medizinischen und sozialen Berufsgruppen, Entscheidungsträger und Institutionen im Gesundheitswesen, Freiwilligen Organisationen, Selbsthilfegruppen und andere.

Die Empfehlungen sind zum Teil konkret und zum Teil offengehalten. Letzteres soll es den Anwendern ermöglichen, die **Umsetzung ihren spezifischen Kontexten und Handlungsmöglichkeiten entsprechend** anzupassen.

Die Entwicklung der Empfehlungen basiert insbesondere auf Daten von Frauen mit chronischen Erkrankungen und Migrationserfahrung; zugleich sind viele der zu Tage gebrachten Ergebnisse **auch für andere Personengruppen anwendbar**. Viele der Verbesserungsvorschläge sind dementsprechend nicht an spezifische Gruppen gebunden, sondern können zur Verbesserung der Versorgungssysteme insgesamt angewendet werden.

Die Empfehlungen definieren zehn Aufgabenbereiche. Dabei sind an verschiedenen Stellen **Überschneidungen zu erkennen**, die u.a. auf deren Bedeutung in verschiedenen Anwendungsfeldern hinweisen.

Die Empfehlungen zielen in erster Linie auf die **Verbesserung von Angeboten der Gesundheitsversorgung** ab. Bei näherem Hinsehen wird zudem deutlich, dass deren Umsetzung positive Wirkungen weit über den engeren Bereich der medizinischen Versorgung haben können und z.B. zur **Förderung des sozialen Zusammenhalts** auf unterschiedlichen Ebenen beitragen können.

Die Empfehlungen sind zurückhaltend bei der Nennung potentiell verantwortlicher **Stellen bzw. AkteurInnen der Umsetzung**. Stattdessen soll Freiraum bleiben, welche Institutionen sich angesprochen fühlen und wo diese Empfehlungen entweder an bestehende Arbeitsbereiche anknüpfen oder als neue Aufgaben wahrgenommen werden können. Die Empfehlungen nehmen keine Stellung zu den Fragen der **Finanzierung der Umsetzung**. Dazu wurden im MIWOCA Projekt keine belastbaren Daten erhoben. Die Hoffnung besteht, dass im Sinne eines solidarischen und fairen Gesundheitswesens bei allen beteiligten AkteurInnen die Bereitschaft vorhanden ist, auch finanzielle Mitverantwortung zu übernehmen.

EMPFEHLUNGEN

1. **Niedrigschwellige Informationsangebote sollen in Gemeinden und Quartieren gefördert werden!**

2. **Der Zugang zu Gesundheitsdienstleistungen soll bei PatientInnen mit chronischen Erkrankungen vereinfacht werden!**

3. **Die Kommunikation zwischen Gesundheitsprofessionen und PatientInnen mit chronischen Erkrankungen soll verbessert werden!**

4. **Bei Erstgesprächen sollen biographische Elemente im Vordergrund stehen!**

5. **Partizipation, Wissen und Kompetenzen von PatientInnen mit chronischen Krankheiten sollen in konkreten Versorgungssituationen gefördert werden!**

6. **Selbsthilfe- und Erfahrungsgruppen sollen gefördert werden!**

7. **Die Regelstrukturen sollen eine integrierte interprofessionelle Versorgung so fördern, dass soziale, psychologische, biomedizinische und Umwelteinflüsse berücksichtigt sind!**

8. **In integrierten Versorgungssystemen soll die Begleitungskompetenz gefördert werden, insbesondere die von Hausärztinnen und Hausärzten!**

9. **Gemeinden sollen ein sozial-unterstützendes und gesundheitsförderliches Umfeld auf Quartiersebene stärken!**

10. **Für Gesundheits- und Sozialberufe sollen mehr und gezieltere Weiter- und Ausbildungsangebote geschaffen werden!**

A) ABBAU VON ZUGANGSBARRIEREN ZU GESUNDHEITSDIENSTLEISTUNGEN

1. **Niedrigschwellige Informationsangebote sollen in Gemeinden und Quartieren gefördert werden!**

↳ Gemeinden bieten auf Quartiersebene PatientInnen mit chronischen Erkrankungen Orientierungshilfen an, insbesondere bei
   - der Suche nach sozialen Diensten, Pflegeangeboten und Selbsthilfegruppen;
   - der Navigation in komplexen Systemen (bspw. in Bezug auf adäquate Versicherungsmodelle und –leistungen);
   - Fragen zu Patientenrechten.

2. **Der Zugang zu Gesundheitsdienstleistungen soll bei PatientInnen mit chronischen Erkrankungen vereinfacht werden!**

↳ Pflegedienste, Ärzte und Ärztinnen stellen definierte Zeitfenster für eine niedrigschwellige Kontaktaufnahme, beispielsweise per Telefon, Messengerdiensten oder Mail, zur Verfügung.

B) FÖRDERUNG VON VERTAUENSBEZIEHUNGEN ZWISCHEN GESUNDHEITSPROFESSIONEN UND PATIENTINNEN

3. **Die Kommunikation zwischen Gesundheitsprofessionen und PatientInnen mit chronischen Erkrankungen soll verbessert werden!**

↳ Bei sprachkompetenzbedingten Verständigungsproblemen zu *komplexen* Fragen stehen *professionelle* Übersetzungsleistungen auch in der ambulanten Versorgung zur Verfügung.

↳ In *einfacheren* Situationen werden vermehrt *digitale* Hilfsmittel eingesetzt, die helfen, die Verständigung zu verbessern (Übersetzungsprogramme).

4. **Bei Erstgesprächen sollen biographische Elemente im Vordergrund stehen!**

↳ Gesundheitsprofessionen berücksichtigen bei der Diagnose und Therapiewahl die Lebenssituation und die gesundheitsrelevanten Kontexte von PatientInnen (inkl. soziale und finanzielle Bedingungen).

↳ Gesundheitsprofessionen erfassen frühzeitig Kompetenzen von PatientInnen und berücksichtigen insbesondere bestehendes Gesundheits- und Krankheitswissen, Körperwahrnehmung oder Copingstrategien.

C) VERBESSERTER EINBEZUG VON PATIENTINNEN UND IHREN RESSOURCEN IN ENTSCHEIDUNGEN ÜBER DIE BEHANDLUNG

5. **Partizipation, Wissen und Kompetenzen von PatientInnen mit chronischen Krankheiten sollen in konkreten Versorgungssituationen gefördert werden!**

↳ Gesundheitsberufe fördern systematisch PatientInnen Wissen und Kompetenzen als Ressource in der Krankheitsbewältigung und damit verbundenen Lebensgestaltung.
↳ Sie stärken damit Gesundheitskompetenz und Selbstmanagement unter Berücksichtigung familiärer und anderer sozialer Unterstützungssysteme.

6. **Selbsthilfe- und Erfahrungsgruppen sollen gefördert werden!**

↳ Mono- und multi-linguale Gruppen werden von Gemeinden und Quartieren gefördert, mit dem Ziel, die Kommunikation und die Unterstützung untereinander zu stärken.
↳ Der Einsatz von sozialen Medien in Selbsthilfeorganisationen und anderen Erfahrungsgruppen wird von öffentlichen Einrichtungen, NGOs, Versicherern, Gemeinden und Quartieren unterstützt.

D) VERBESSERUNG DER ZUSAMMENARBEIT ZWISCHEN GESUNDHEITSBERUFEN

7. **Die Regelstrukturen sollen eine integrierte interprofessionelle Versorgung so fördern, dass soziale, psychologische, biomedizinische und Umwelteinflüsse berücksichtigt sind!**

↳ Darin wird die Diversität von Bedarfen und Bedürfnissen der PatientInnen verstanden, anerkannt und die Praxis danach ausgerichtet.
↳ Aktuelle Listen der relevanten Versorger im Gesundheits- und Sozialbereich mit entsprechenden Sprachkompetenzen stehen leicht zugänglich zur Verfügung; bereits existierende Listen werden proaktiver als bisher verteilt.

8. **In integrierten Versorgungssystemen soll die Begleitungskompetenz gefördert werden, insbesondere die von Hausärztinnen und Hausärzten!**

↳ Spezifische Ausbildungs- und Weiterbildungsangebote dazu werden konzipiert und angeboten.
↳ Sozialarbeitende unterstützen Ärztinnen und Ärzte, um die sozialen, ökonomischen und migrationsbedingten Einflüsse in die therapeutischen Entscheide mit einzubeziehen.

E) FÖRDERUNG DES NICHT MEDIZINISCHEN UNTERSTÜTZENDEN UMFELDS

9. Gemeinden sollen ein sozial-unterstützendes und gesundheitsförderliches Umfeld auf Quartiersebene stärken!

- ↳ Die soziale Inklusion von PatientInnen mit chronischen Erkrankungen wird gezielt gefördert.
- ↳ Speziell in der Unterstützung von Personen mit chronischen Krankheiten stehen Anlaufstellen zur Verfügung und informieren über und unterstützen kulturelle und soziale Partizipationsmöglichkeiten und fördern die Freiwilligenarbeit.

F) VERBESSERTE WEITER- UND AUSBILDUNGSANGEBOTE

10. Für Gesundheits- und Sozialberufe sollen mehr und gezieltere Ausbildungsangebote geschaffen werden!

- ↳ Spezifische Aus- und Weiterbildungen werden von den etablierten Ausbildungsinstitutionen (unter anderem Universitäten, Spitäler und NGOs) entwickelt und zur Verfügung gestellt. Zentrale Themen dabei sind: «(Inter-) Professioneller Umgang mit sozialen und administrativen Herausforderungen von Personen mit chronischen Krankheiten», und «Transkulturelle und transkategoriale Herangehensweisen in der Arbeit mit chronisch kranken PatientInnen».
