# Supplementary material for: Integrating Patient‐reported Experience (PRE) in a multistage approach to study access to health services for women with chronic illness and migration experience
Source: Health Expect. 2022 Nov 23;26(1):237–44. doi: 10.1111/hex.13649 (PMC9854289; doi:10.1111/hex.13649)
Supplement: Supplementary file 3 — Additional File 3. [file HEX-26--s001.pdf]

74 NRP Smarter Health Care
National Research Programme

SocioBrief
2020/N° 3A

Improving Health Care for Women with Chronic Illness
Focus on Migrant Women (MIWOCA)

Annika Frahsa, Thomas Abel, Tevfik Bayram, Sandro Cattacin, Luna de Araujo, Romaine Farquet, Sophie Meyer and Sibel Sakarya

Key messages

- MIWOCA presents 10 recommendations for improving health care for women with chronic conditions, with special consideration of women with migration experience.
- These recommendations are based on interviews and focus groups with women, interviews with health care providers, as well as dialogues between them and health specialists and decision makers.
- MIWOCA identified barriers and resources to access health care, by focusing on Patient Reported Experiences (PRE).

Summary

In Switzerland, access to health care is limited for various population groups. For example, women with migration background use some health care services (e.g. early detection measures) less frequently than the rest of the population, even though they suffer more frequently from chronic conditions. In view of these differences, the MIWOCA project analysed women's experiences with the health care system in the Cantons of Bern and Geneva, the barriers they faced and the resources they used to access high quality care.

Women from Germany, Portugal, Turkey and Switzerland participated in various parts of the project. Complementary interviews with health and social services providers were also conducted. During interviews and focus groups, the women highlighted several issues that affect them, and named their resources and strategies to overcome the barriers faced. These might include becoming experts of their own illness over time or using their social network's support.

During stakeholder dialogues - the last phase of the project, the women shared their experiences and needs with relevant health and social services specialists. Together, they developed 10 recommendations to improve access to healthcare.

MIWOCA
Migrant Women's Health Care Needs for Chronic Illness Services in Switzerland

MIWOCA is part of the National Research Programme 74, funded by the Swiss National Science Foundation. It is coordinated by the University of Bern (Prof. T. Abel, ISPM), in collaboration with experts from universities in Geneva, Tübingen and Istanbul.

Full recommendations

www.miwoca.ch

Further readings

www.bag.admin.ch

Main results of the second monitoring of the health status of the migrant population in Switzerland (GMM II), Federal Office of Public Health, 2010

SocioBrief is a briefing note based on scientific work, the aim of which is to shed light on contemporary debates on societal issues.
SocioBrief is aimed at a professionals active in public policy and related fields of action.

Results

Descriptive data show that the burden of chronic diseases is high in Switzerland, particularly among migrant women (GMM II), and low health literacy as well as a potential over- or undersupply of services prevail among certain migrant groups. At the same time, little is known about how women with chronic illness and migration experience use health care in Switzerland. To date, the practical experience of affected patients has rarely been included in the description of the problem, and the women themselves are hardly involved in the development of solutions.

The MIWOCA project builds on Sen's pioneering capability approach to explore Patient Reported Experiences (PRE) with Swiss health services among women with chronic conditions and migration experience.

MIWOCA is a multi-method qualitative study. 48 semi-structured interviews were conducted with women in Bern and Geneva: 12 women from Germany, 12 from Turkey, 12 from Portugal and 12 from Switzerland. The participants were heterogeneous in terms of age, length of stay in Switzerland, socio-economic and educational situation, type of illness and medical history. Two focus groups with interviewed women were also hosted. In addition, 12 complementary semi-structured interviews were conducted with healthcare providers. The results obtained describe the actual conditions (problems, barriers, resources) from the patients' (i.e. the users') perspective.

The women highlighted issues such as communication problems, distrust in healthcare professionals, stereotyping, lack of understandable information on the healthcare and insurance systems, lack of coordination between healthcare providers, high healthcare-related services costs or difficulties in finding a family doctor and other therapists. The women's resources and strategies to face these barriers included becoming expert of their own illness over time, reducing communication problems by learning local language and medical terms, relying on social networks to find a doctor, for translations or for emotional, administrative and financial support.

The results were incorporated into three stakeholder dialogues in which women, health care providers and political decision-makers from different levels participated. The stakeholder dialogues developed 10 practical recommendations for improving health care in Switzerland for women, presented below.

Recommendations

1. **Low-threshold information services should be promoted in municipalities and neighbourhoods.**
2. **Access to health services should be simplified for patients with chronic illness.**
3. **Communication between health professionals and patients with chronic illness should be improved.**
4. **Biographical elements should be in the foreground during initial consultations.**
5. **Participation, knowledge, and competences of patients with chronic illness should be promoted in concrete care situations.**
6. **Self-help and other support groups should be promoted.**
7. **Regulatory structures should promote integrated inter-professional care so that social, psychological, biomedical, and environmental factors are accounted for.**
8. **In integrated care systems, competencies on ensuring continuity of care should be promoted, especially for family doctors.**
9. **Municipalities should strengthen a socially supportive and health-promoting environment at the neighbourhood level.**
10. **Health and social professionals should be offered more and specifically-targeted training opportunities.**

Annika Frahsa et al. *Improving Health Care of Women with Chronic Illness. Focus on Migrant Women*, Genève : Université de Genève (SocioBrief, n°3a)
The sole responsibility for the content of this document rests with its authors. ISSN : 2673-2742

UNE PUBLICATION DE L'INSTITUT DE RECHERCHES SOCIOLOGIQUES
FACULTÉ DES SCIENCES DE LA SOCIÉTÉ
